# Care pathways models and clinical outcomes in Disorders of consciousness

**DOI:** 10.1002/brb3.740

**Published:** 2017-07-21

**Authors:** Davide Sattin, Laura Morganti, Laura De Torres, Giuliano Dolce, Francesco Arcuri, Anna Estraneo, Viviana Cardinale, Roberto Piperno, Elena Zavatta, Rita Formisano, Mariagrazia D'Ippolito, Claudio Vassallo, Barbara Dessi, Gianfranco Lamberti, Elena Antoniono, Crocifissa Lanzillotti, Jorge Navarro, Placido Bramanti, Francesco Corallo, Mauro Zampolini, Federico Scarponi, Renato Avesani, Luca Salvi, Salvatore Ferro, Luigi Mazza, Paolo Fogar, Sandro Feller, Fulvio De Nigris, Andrea Martinuzzi, Mara Buffoni, Adriano Pessina, Paolo Corsico, Matilde Leonardi

**Affiliations:** ^1^ Neurology, Public Health, Disability Unit – Scientific Department Fondazione IRCCS Istituto Neurologico Carlo Besta Milan Italy; ^2^ RAN (Research in Advanced Neurorehabilitation) – Istituto S. Anna Crotone Italy; ^3^ Disorders of Consciousness Laboratory Salvatore Maugeri Foundation IRCCS Scientific Institute of Telese Terme Telese Terme Italy; ^4^ Neurorehabilitation Unit Emergency Department AUSL of Bologna Bologna Italy; ^5^ Centro Studi per la Ricerca sul Coma ‐ “Gli Amici di Luca” ONLUS Casa dei Risvegli Luca De Nigris Bologna Italy; ^6^ Unità Post‐Coma IRCCS Fondazione Santa Lucia Roma Italy; ^7^ Dipartimento di Psicologia Università “La Sapienza” Roma Italy; ^8^ Centro di Riabilitazione Ambulatoriale Associazione Rinascita Vita ONLUS Genova Italy; ^9^ S.C. Neuroriabilitazione ASL CN1 Ospedale “SS. Trinità” ‐ Fossano Fossano Italy; ^10^ Fondazione San Raffaele ‐ Presidio Ospedaliero di Ceglie Messapica Ceglie Messapica Italy; ^11^ IRCCS Centro Neurolesi “Bonino‐Pulejo” Messina Italy; ^12^ Neurorehabilitation Unit “S.Giovanni Battista” Hospital Foligno Italy; ^13^ Dipartimento di Riabilitazione Ospedale Sacro Cuore Don Calabria Verona Italy; ^14^ Emilia Romagna Region Direzione Generale Cura della Persona, Salute e Welfare Bologna Italy; ^15^ Emilia Romagna Region Servizio Integrazione Sociosanitaria e politiche per la Non Autosufficienza Bologna Italy; ^16^ Federazione Nazionale Associazioni Trauma cranico Carnago Italy; ^17^ “La rete” Association (Amici di Luca onlus) Bologna Italy; ^18^ IRCCS Medea Conegliano Research Centre Conegliano Italy; ^19^ Bioethics University Centre Università Cattolica del Sacro Cuore Milan Italy

**Keywords:** disorders of consciousness, health services research, long‐term care, rehabilitation outcomes, vegetative state

## Abstract

**Objective:**

Patients with Disorders of consciousness, are persons with extremely low functioning levels and represent a challenge for health care systems due to their high needs of facilitating environmental factors. Despite a common Italian health care pathway for these patients, no studies have analyzed information on how each region have implemented it in its welfare system correlating data with patients’ clinical outcomes.

**Materials and Methods:**

A multicenter observational pilot study was realized. Clinicians collected data on the care pathways of patients with Disorder of consciousness by asking 90 patients’ caregivers to complete an ad hoc questionnaire through a structured phone interview. Questionnaire consisted of three sections: sociodemographic data, description of the care pathway done by the patient, and caregiver evaluation of health services and information received.

**Results:**

Seventy‐three patients were analyzed. Length of hospital stay was different across the health care models and it was associated with improvement in clinical diagnosis. In long‐term care units, the diagnosis at admission and the number of caregivers available for each patient (median value = 3) showed an indirect relationship with worsening probability in clinical outcome. Caregivers reported that communication with professionals (42%) and the answer to the need of information were the most critical points in the acute phase, whereas presence of Non‐Governmental Organizations (25%) and availability of psychologists for caregivers (21%) were often missing during long‐term care. The 65% of caregivers reported they did not know the UN Convention on the Rights of Persons with Disabilities.

**Conclusion:**

This study highlights relevant differences in analyzed models, despite a recommended national pathway of care. Future public health considerations and actions are needed to guarantee equity and standardization of the care process in all European countries.

## 1 INTRODUCTION

Patients in vegetative state (VS) or in minimally conscious state (MCS), generally grouped in the term Disorders of Consciousness (DOC), are clinically classified as unconscious or low‐responsive patients, respectively, and unable, or only partially able, to communicate their feelings and experiences (Bernat, 2006). In Italy, the Italian Ministry of Health technical report on patients with DOC (Stato Vegetativo e di Minima Coscienza ‐ Epidemiologia, evidenze scientifiche e modelli assistenziali, http://www.salute.gov.it/imgs/C_17_pubblicazioni_1378_allegato.pdf, Accessed June 20, 2016.) reported rates of incidence and prevalence of 0.5–4/100.000 and 0.6–10/100.000, respectively, although these numbers seem to underestimate the real increasing incidence of VS and MCS in Italy as reported in the same document. Moreover, the incidence and prevalence of DOC is increasing for several epidemiological reasons, for example, aging of population (associated with the incidence of severe vascular brain injury) and the improvement in clinical management that determines an increase in the survival rate (Donis & Kraftner, 2011; Higashi et al., 1977; Lavrijsen, van den Bosch, Koopmans, & van Weel, 2005; Pisa, Biasutti, Drigo, & Barbone, 2014; Saout et al., 2010; Stepan, Haidinger, & Binder, 2004).

Persons with DOC require several treatments and usually have a long hospital stay. Considering the severe cognitive and motor disabilities and the absence of functional communication, professionals must be specifically trained for management of patients with DOC and a tailored care pathway is required to guarantee adequate clinical management, promoting patients’ safety, supporting recovery of consciousness, and optimizing public health costs.

However, only some European nations published guidelines defining care pathways for patients with DOC (Cuadernos fedace sobre daño cerebral adquirido: síndrome de vigilia sin respuesta y de mínima conciencia, http://fedace.org/wp-content/uploads/2013/09/13_vigilia_conciencia.pdf, Accessed June 28, 2016; Prolonged disorders of consciousness: national clinical guidelines, https://www.rcplondon.ac.uk/guidelines-policy/prolonged-disorders-consciousness-national-clinical-guidelines, Accessed May 12, 2016; Godbolt, Tengvar, Johansson, Stenson, & Borg, 2011; Ministère de la Santé et de la Protection Sociale, Secrétariat d'Etat aux personnes handicapées, 2002; von Wild et al., 2007). Italy is one of them and, in 2009, the Ministry of Health convened a technical committee, composed by professionals and Non‐Governmental Organizations (NGOs) representing caregivers of patients with DOC, that wrote the first national pathway of care model for patients with diagnosis of VS and MCS (from now NPCM‐DOC; Stato Vegetativo e di Minima Coscienza ‐ Epidemiologia, evidenze scientifiche e modelli assistenziali, http://www.salute.gov.it/imgs/C_17_pubblicazioni_1378_allegato.pdf, Accessed June 20, 2016; see Supporting Information for details). However, NPCM‐DOC specified only the aims of the different phases of care for patients with DOC, reporting general characteristics of services to be provided in each step without indicating how each “box” of the diagram should be implemented in the country. Italy is composed of 20 regions and all of them implement national rules according to their regional welfare characteristics, causing differences in health care pathways from one region to another. For example, there are Italian regions that have subsidized the home care provision through social allowances for chronic patients, while other regions have been increasing public centers’ services. Obviously, different application of the same national model could cause different effects, such as different length of stay (LOS) in hospital units, different numbers in hospital admissions, and different possibilities for long‐term care assistance.

Taking into account the bio‐psycho‐social model proposed in the International Classification of Functioning, Disability and Health (ICF) of the World Health Organization (WHO; World Health Organization, 2001), information on the effect of environmental factors, such as those related to care pathways, is particularly important especially if they impact directly on patients’ clinical outcomes or caregivers distress (Giovannetti, Covelli, Sattin, & Leonardi, 2015). To the best of our knowledge, no studies have systematically analyzed this effect for patients in VS and MCS at the moment, and information on relationships between care pathways and clinical outcomes are still lacking. This theme is also particularly important for ethical and legal issues related to the management of persons with DOC also considering the knowledge and role of caregivers during the care process of persons unable of self‐determination.

Therefore, the aim of the present multicentric study was twofold: first, it aimed to analyze current NPCM‐DOC implementation within different regional healthcare models focusing on the relationship between characteristics of care process with patients’ clinical outcomes; second, to identify caregivers’ opinions for each phase of the care process paying attention to critical points they noticed.

## 2 MATERIALS AND METHODS

A multicenter observational study involving 10 intensive rehabilitation centers in different Italian Regions was conducted between November 2012 and November 2014. This study on care pathways for persons with disorders of consciousness was approved by coordinator Ethical Committee and was performed in accordance with the Declaration of Helsinki. Written informed consent was obtained from all caregivers’ legal representative of all patients.

### 2.1 Procedure

From October 2013 to May 2014, clinicians from each participating rehabilitation center contacted the main informal caregivers of all patients discharged with a diagnosis of DOC after traumatic or nontraumatic acute event (evaluated according to the Aspen criteria Giacino et al. (1977) and the American Congress of Rehabilitation Medicine, Brain Injury‐Interdisciplinary Special Interest Group, Disorders of Consciousness Task Force (2010)) from their units in 2011. In according to the regional models of care, participating centers could have only one unit (post acute rehabilitation unit) or two (sub acute and post acute rehabilitation units) in the same hospital. In this last case, in the present study we included all patients discharge from both units in 2011.

During phone calls, researchers asked the caregivers if he/she wanted to participate in the INCARICO project completing a structured phone interview in order to collect data on the care pathways of patients with DOC and collecting his/her opinions on the services provided to the patient he/she care for (main informal caregiver was defined highlighting the concept of assuming responsibility for the person who needs help as specified by Gould (2004)). If they accepted, researchers sent them the written informed consent and when caregivers sent it back to clinicians, professionals called again the caregivers and began the phone interview.

An ad hoc questionnaire (see Supporting Information) was developed for the INCARICO Project‐phone interview. The questionnaire was developed considering data and results from national reports derived from previous researches (Giovannetti, Cerniauskaite, Leonardi, Sattin, & Covelli, 2015). It was developed taking into consideration NGOs frequently asked questions collected by caregivers on care pathways (Libro bianco sugli Stati Vegetativi e di Minima Coscienza. Il punto di vista delle Associazioni che rappresentano i familiari, http://www.salute.gov.it/imgs/C_17_pubblicazioni_1377_allegato.pdf, Accessed April 25, 2017). If a caregiver did not respond to a question, or said “I don't know”, interviewer proposed different issues related to services, professionals, and personal judgments for each clinical pathway phase. The “Not applicable” response was also possible when the service reported in the item was not provided for patients during the care process. If a patient died before the interview, caregiver could choose to complete the questionnaire or not, considering their emotional state. All interviewers asked the caregiver to complete the questionnaire after consulting all medical records available for clinical variables (e.g., hospitalization dates, last diagnosis, complete name of the institutions, etc.). For patients at home, the last diagnosis was collected considering the last medical records reported by multidisciplinary teams who evaluated clinical status of each patient every year.

Main variables derived from the questionnaire and analyzed in this study were: Length of Stay (LOS), Transitions along Phases (TP), Changes in Diagnosis (CD) and Mortality, Adherence to regional pathway of care (APC), number of caregivers available for patient assistance (nCG). LOS was calculated by summing the number of days spent by a patient in intensive care unit (ICU) and rehabilitation units in order to know how long a patient was hospitalized in health care service centers, noting also the first admission in ICU and RH. In this study, the variable “LOS in SA+RH” was obtained considering LOS in subacute and postacute units. TP consists of the number of admissions to health centers for each patient during his care pathway. For example, the TP of a patient who was admitted in three intensive care units, two semi‐intensive care units, three rehabilitation units, and one long‐term care center during his/her care process was 9. CD represents the number of changes in patient's diagnosis registered along the care process. In particular, this variable indicates the improving or the worsening in clinical diagnosis during hospitalization. In this study, comparison between the diagnosis at discharge after the first hospitalization in a rehabilitation center and the final one (at the moment of the interview) was used as an outcome measure. APC was calculated considering the percentage of patients who completed their cure and care process in the same region of residence. In detail, for each region, the number of patients who lived in that region and completed their care process in the same region was calculated and the number of patients who were hospitalized in that region but with their home residence in another region was also considered. nCG is the number of persons reported by caregivers who participated in caring process of one patient.

### 2.2 Statistical analysis

Data derived from INCARICO questionnaire were analyzed in accordance with the following statistical methods: nominal variables are presented as number or percentage, continuous variables are presented as mean and standard deviation (*SD*) or median and interquartile range (IR) or minimum‐maximum range. Non‐normal distribution of the continuous variable was verified using the Kolmogorov–Smirnov test and analysis of skewness and kurtosis. Bootstrap method was used and confidence intervals were obtained with the “BCA” (bias corrected and accelerated) variations at 1,000 resamples for total median values reported in Table 1.

**Table 1:** Differences in hospital Length of stay (acute and postacute phase), number of interhospital patient transfers, and data on adherence to regional care pathway among Italian regions analyzed

Italian Regions	No. of patients^a^	ICU LOS (first hospitalization)	ICU LOS (total)	Subacute LOS^b^	Rehabilitation LOS	LOS Ratio	Number of interhospital patient transfers	APC	Patients hospitalized from other regions	Mortality
	*n*	Median (IR)	Median (IR)	Median (IR)	Median (IR)	Median (IR)	Median (IR)	*n* (%)	*n* (%)	*n* (%)
Calabria	10	37.5 (70)	58.5 (81)	209.5 (152)	72 (233)	175 (158.25)	5.5 (2)	8 (80.0)	0 (0)	1 (10)
Campania	12	43 (46)	52.5 (46)	177.5 (195)	8 (146)	174 (104.75)	4 (5)	7 (58.3)	1 (6.7)	3 (20)
Emilia Romagna	9	30 (25)	101 (101)		210 (315)	47.6 (49.48)	12 (5)	4 (44.4)	4 (44.4)	0 (0)
Lazio	5	77 (76)	133 (127)		503 (340)	197.33 (197.05)	5 (5)	4 (80)	1 (20.0)	0 (0)
Liguria	6	37 (143)	48.5 (131)	10.5 (181)	182 (195)	147.62 (95.88)	6 (3)	4 (66.7)	1 (16.7)	4 (66.7)
Piedmont	6	102.5 (97)	102.5 (97)	0 (0)	368 (629)	259.25 (628.75)	3 (0)	6 (100)	0 (0)	2 (33.3)
Apulia	5	50 (80)	50 (106)	127 (263)	122 (156)	126 (125.25)	5 (3)	3 (60.0)	0 (0)	9 (64.3)
Sicily	6	43 (41)	43 (41)	373 (470)	0 (58)	334.5 (470)	2 (1)	5 (83.3)	0 (0)	0 (0)
Umbria	7	43 (93)	66 (93)	0 (0)	179 (303)	135.66 (93)	3 (2)	4 (57.1)	2 (16.7)	5 (41.7)
Veneto	7	61 (30)	74 (59)	168 (356)	143 (182)	112.66 (236.5)	5 (2)	4 (57.1)	1 (14.3)	0 (0)
Tot.	73	44	62	56	142		5	49 (67.1)	10 (13.6)	24 (26.6)^c^
Lower‐upper boundary^d^		(39–57)	(50–74)	(0–139)	(110–174)		(4–6)			

ICU, intensive care units; LOS, length of stay; LOS RATIO, proportion between total number of LOS in subacute and rehabilitation units (total number of days spent in subacute and rehabilitation units) and number of admission in rehabilitation units for each patient (median number of LOS each admission); APC, adherence to regional care process (patients who complete their cure and care process in the same region of residence).

a Patients included in data analysis.

b Subacute units are not activated in all regions.

c Percentage was calculated considering the total sample of 90 patients included in the project.

d The range of the median boundaries were obtained from 1,000 bootstrap replicates.

Series of univariate binary logistic regression analysis were performed to test relationships between each variable described in the previous section with outcome. Analysis details are reported in Supporting Information. All data were analyzed using SPSS 18.0 software (SPSS Inc., Chicago, IL, USA).

## 3 RESULTS

Ninety patients were discharged from the participating centers in 2011. Of these, 24 (26.7%) died before the phone call interview as reported by their caregivers but seven of them decided to complete the INCARICO questionnaire in any case (Fig. S1).

At the time of death, the majority of them (no. 17, 73.9%) were hospitalized in nursing home, except one in hospital, whereas 6 (26.1%) were at home. In Table 1, the percentage of dead patients across regions are reported. Survivors were mainly males (no. 54, 60%), mean age was 50.5 (±18.8) years and mean time from acute event was 42.7 (±19.4) months. Regarding the mean age of patients who were hospitalized, the youngest (median age 26, min 24–max 66 years old) were from the Lazio region, whereas the oldest ones (median age 69.5, min 52–max 82 years old) were from the Sicily region.

Forty‐six patients (54.8%) had MCS diagnosis at the time of the interview, whereas 29 (34.5%) were in VS and 9 (10.7%) were emerged from MCS remaining with a severe disability. For six patients, caregivers did not report diagnosis (all patients were dead before the interview). Changes in diagnosis were found mainly during hospitalization in the rehabilitation centers where 29 patients (39.7% of 73 patients analyzed) improved from VS to MCS, and 8 (10.9%) from MCS to Severe disability diagnosis, whereas 7 (9.5%) patients had a change in their diagnosis during long‐term phase. Data collected on acute and subacute phases are reported in Table 1. Fifteen (20%) patients were re‐admitted in rehabilitation units after their return at home or hospitalization in nursing homes.

Results from multinomial logistic regression analysis for acute and rehabilitation phase are reported in Table 2. No statistically significant *p*‐values were found for the general model which included all independent variables both with forced and backward stepwise methods (first and second analysis steps). Interaction between LOS in RH and time from acute event or age showed low *R* ^2^ values but statistically significant. LOS was statistically significant both in the first (model A) and the second model (model B); so, the probability of finding changes in odds ratio in clinical diagnosis were directly proportionally to number of days spent in subacute/rehabilitation units by patients, so more days corresponded to greater probability of an improvement in clinical status (change from VS to MCS or from it to Severe Disability) than a worsening. No other variables than LOS seemed statistically significant in predicting the probability of a clinical status improvement than a worsening in our study.

**Table 2:** Multinomial Multivariate Logistic analysis. Relationships between improvement in clinical status and variables related to acute and postacute phases

		Independent variables						
		Time from acute event	Age	LOS – ICU	LOS – SA+RH	APC	TP	
Model A								
Clinically stable vs. improving								*R* ^2^ = .179 (Cox and Snell), .202 (Nagelkerke); Model χ^2^(2) = 16.163, *p* = .003**
B (*SE*)	−442 (0.962)				−0.001 (0.001)			
OR (95% CI)			1.010 (0.981–1.040)		0.999 (0.997–1.001)			
Sig.			.516		.383			
Clinical Worsening vs. improving								
B (*SE*)	0.998 (964)		0.003 (0.15)		−0.005 (0.002)			
OR (95% CI)			1.003 (974–1.034)		0.995 (0.992–0.998)			
Sig.			.826		.001**			
Model B								
Clinically stable vs. improving								*R* ^2^ = .128 (Cox and Snell), .144 (Nagelkerke); Model χ^2^(2) = 10.509, *p* = .033*
B (*SE*)	0.392 (0.840)		−0.009 (0.022)		−0.001 (0.001)			
OR (95% CI)			0.991 (0.950–1.035)		0.999 (0.996–1.002)			
Sig.			.688		.496			
Clinical Worsening vs. improving								
B (*SE*)	0.922 (0.878)		−0.001 (0.023)		−0.004 (0.002)			
OR (95% CI)			0.999 (0.954–1.046)		0.996 (0.992–0.999)			
Sig.			.956		.011*			

LOS‐ICU, length of stay in intensive care units; LOS SA+RH, length of stay in subacute and rehabilitation units; APC, patients who complete their cure and care process in the same region of residence; TP, number of hospitalization for each patient during his care process. Clinical worsening vs improving: the probability to have a decrease in patient diagnosis during post acute hospitalization rather than the probability to have an increase in clinical status (y=improving).

*Predictors statistically significant in the model: *p* < .05; **p < .01; ***p < .0001

Table 3 shows the results of multinomial regression analysis for long‐term care phase. Model with all independent variables included showed a *R* ^2^ greater than those after backward stepwise methods. Diagnosis of MCS or severe disability after rehabilitation phase significantly increase the probability of finding an improvement in patient outcome/diagnosis in long‐term care center (or at home) rather than a worsening (odd ratio increase of 10.636 and 9.391 in the first (model C) and the second model (model D), respectively). In the same way, the number of persons who cared for the patients represented a significant variable in predicting clinical improvement (than worsening) independently of the fact whether the patients were admitted in nursing home or at home.

**Table 3:** Multinomial Multivariate Logistic analysis. Relationship between the probability of worsening in clinical status and variables related to long‐term care phase

		Independent variables					
		Diagnosis	nCG	Time from acute event to admission in nursing home/home	Transfer from nursing home/home to ICU	Last admission	
Model C							
Clinical improving vs. Worsening							*R* ^2^ = .358 (Cox and Snell), .408 (Nagelkerke); Model χ^2^(2) = 22.181, *p* = .014*
B (*SE*)	−2.413 (1.638)	2.364 (1.039)	0.973 (0.444)	0.047 (0.060)	−0.266 (0.705)	−0.433 (1.318)	
OR (95% CI)		10.636 (1.389–81.433)	2.646 (1.108–6.322)	1.048 (0.932–1.178)	0.766 (0.192–3.052)	0.649 (0.049–8.591)	
Sig.		.023*	.029*	.435	.706	.743	
Clinically stable vs. Worsening							
B (*SE*)	0.761 (1.081)	−0.060 (0.895)	0.739 (457)	−0.035 (0.056)	−0.820 (0.831)	−0.828 (1.170)	
OR (95% CI)		0.942 (0.163–5.444)	2.093 (0.855–5.123)	0.966 (0.866–0.1.078)	0.440 (0.086–2.243)	0.437 (0.044–0.325)	
Sig.		.946	.106	.537	.323	.479	
Model D							
Clinical improving vs. Worsening							*R* ^2^ = .303 (Cox and Snell), .344 (Nagelkerke); Model χ^2^(2) = 18.026, *p* = .001**
B (*SE*)	−2.107 (1.030)	2.240 (1.013)	0.877 (0.399)				
OR (95% CI)		9.391 (1.290–68.347)	2.403 (1.099–5.256)				
Sig.		.027*	.028*				
Clinically stable vs. Worsening							
B (*SE*)	−0.123 (725)	−0.365 (0.818)	0.595 (0.399)				
OR (95% CI)		0.694 (0.140–3.448)	1.813 (0.829–3.963)				
Sig.		.655	.136				

Diagnosis, diagnosis at discharge from rehabilitation units (other diagnosis vs. Vegetative State diagnosis); nCG, number of caregivers available for patient assistance; ICU, intensive care units; Last admission, last place in which patients were admitted for long‐term care (home vs. nursing home); Clinical improving vs worsening: the probability to have an increase in patient diagnosis during long‐term care rather than the probability to have a worsening in clinical status (y = worsening).

*Predictors statistically significant in the model: *p* < .05; **p < .01; ***p < .0001.

Seventy caregivers of 73 patients participated in the last part of the interview (3 caregivers of 7, whose patients were dead before interview did not complete the last part of the questionnaire). Their mean age was 54.7 years (±11.8), 40 (57.2%) were female and mean time dedicated to patient assistance was 13.5 hr/day (±8.1) at the moment of the interview. The median number of caregivers per patients was 3 (IR 1) and main caregiver was usually a patients’ relative (wife 22.9%, mother 21.4%, sons 20%, husband 14.3%, father 12.9%, brother/sister 2.9%), cohabitant (1.4%), or persons paid for caring (4.2%). Results on caregivers’ opinions are reported in Table 4. Fourteen caregivers did not complete questionnaire related to the long‐term care phase either because patients died during postacute phase or because they were still hospitalized in rehabilitation centers. The section relative to long‐term care phase was completed by 56 caregivers (43 patients were at home, whereas 13 in nursing homes).

**Table 4:** Items evaluated as strong or critical points by caregivers for the different phases of healthcare pathway

	Strong point	Weak point	Not evaluated	N/A	No. of respondents, *n* (%)
Acute phase					
Communication modalities and information completeness	34 (48.57)	30 (42.86)	6 (8.57)	—	70 (100)^a^
Quantity of the received healthcare services	41 (58.57)	24 (34.29)	5 (7.14)	—	70 (100)
Quality of the received healthcare services	38 (59.38)	23 (35.94)	3 (4.69)	—	64 (100)
Decision about the center for the next phase of care	35 (50.72)	23 (33.33)	11 (15.94)	—	69 (100)
Waiting time for admission in the center of the next phase of care	42 (60)	19 (27.14)	9 (12.86)	—	70 (100)
Postacute phase (Rehabilitation)					
Center's reception modalities	58 (85.29)	10 (14.71)	0 (0)	—	68 (100)
Communication modalities and completeness of information	56 (82.35)	12 (17.65)	0 (0)	—	68 (100)
Visiting policies	55 (80.88)	12 (17.65)	1 (1.47)	—	68 (100)
Psychologist	35 (50.72)	17 (24.64)	11 (15.94)	6 (8.7)	69 (100)
Social worker	45 (65.22)	11 (15.94)	8 (11.59)	5 (7.25)	69 (100)
Nongovernment associations	29 (42.02)	20 (28.99)	20 (28.99)	—	69 (100)^a^
Setting	61 (89.71)	4 (5.88)	3 (4.41)	—	68 (100)
Decision about the center for the next phase of care	41 (60.29)	17 (25)	10 (14.71)	—	68 (100)
Waiting time for admission in the center of the next phase of care	37 (54.41)	9 (13.24)	22 (32.35)	—	68 (100)
Rehabilitation service's quality	56 (81.16)	11 (15.94)	2 (2.9)	—	69 (100)
Rehabilitation service's quantity	50 (72.46)	17 (24.64)	2 (2.9)	—	69 (100)
Long‐term care phase					
Center's reception modalities	32 (57.14)	4 (7.14)	20 (35.72)	—	56 (100)
Communication modalities and completeness of information	37 (66.07)	12 (21.42)	7 (12.51)	—	56 (100)
Visiting policies	27 (48.21)	5 (8.92)	3 (5.35)	21 (37.52)	56 (100)
Presence of Psychologist for caregiver	19 (33.92)	12 (21.42)	15 (26.78)	10 (17.88)	56 (100)^a^
Presence of Social worker	27 (48.21)	12 (21.42)	4 (7.14)	13 (23.23)	56 (100)
Nongovernment associations	16 (28.57)	14 (25.01)	26 (46.42)	—	56 (100)^a^
Setting	28 (50.0)	5 (8.92)	23 (41.08)	—	56 (100)
Assistance in case of urgency/emergency	42 (75.00)	4 (7.14)	4 (7.14)	6 (10.72)	56 (100)
Possible readmission in the same nursing home after hospitalization in other units	19 (33.92)	5 (8.92)	4 (7.14)	28 (50.02)	56 (100)
Possible readmission in rehabilitation centers	35 (62.50)	3 (5.35)	4 (7.14)	14 (25.01)	56 (100)
Care service's quality	35 (62.50)	11 (19.64)	10 (17.86)	—	56 (100)
Care service's quantity	31 (55.35)	16 (28.57)	9 (16.08)	—	56 (100)

N/A, Not Applicable.

a Percent (%) of “weak point” plus “not evaluated” responses > strong point response.

Regarding caregivers’ knowledge of the UN Convention on the Rights of Persons with Disabilities, 65.7% of caregivers reported that they did not know it, and 7.1% said they knew it only partially. The national agreement on VS patients care pathway signed between each regional authority and Ministry of Health was known by 25.7% of the sample.

## 4 DISCUSSION

We, analyzed health care pathways in a group of 90 patients with Disorders of Consciousness hospitalized in, and discharged by, 10 rehabilitation centers in 2011, in different regions of Italy. Results can be broadly divided in two areas: analysis of the differences in care process for patients with DOC and clinical outcomes, and analysis of caregivers’ opinions. In the first area, results showed that LOS in ICU seems to be quite homogeneous among the different regional models, although ICU in Lazio and Piedmont models showed higher values than other regions, whereas LOS in subacute and rehabilitation units appeared more variable, ranging from 50 to more than 300 days/hospitalization (median values). The relationship between LOS and number of admission in rehabilitation units seemed to highlight three different general models across regions (considering total LOS in RH and LOS/number of admission in RH ratio): Emilia Romagna showed a care process characterized by short hospitalizations (around 2 months) but repeated along care process (model 1); Lazio, Piedmont, and Sicily models reported fewer admissions in RH units but with higher LOS values than the other regions, ranging from 6 months to 1 year (model 2); and Calabria, Campania, Liguria, Apulia, Umbria, and Veneto models showed a median LOS in RH value ranging from 4 to 6 months (model 3). The analysis on the relationship between LOS variables in the acute and postacute phases of care process and improvement in clinical diagnosis showed that LOS in ICU did not seem to be really related to probability in diagnostic improvement in our sample. The higher LOS values in RH could be partially related to the age of patients (e.g., in Lazio region, hospitalized patients were younger than those in other regions), and to the availability of sufficient number of rehabilitation units or chronic facilities to ensure continuing care in appropriate setting and/or related to clinical severity.

LOS in RH, instead, appeared to be a statistically significant variable for improvement in clinical outcome although its effect was relatively low. Long‐term rehabilitation process, for instance, could be useful to intercept early signals of consciousness recovery by skilled professional experienced in the accurate and standardized clinical assessment (Estraneo et al., 2015; Willems, Sattin, Vingerhoets, & Leonardi, 2015), although “late recovery” after the classical temporal limits of 6 and 12 months post nontraumatic and traumatic brain injury, respectively, could not be clearly related to LOS in RH (Estraneo, Moretta, Loreto, Santoro, & Trojano, 2014). The possibility of one or more readmission in RH units during long‐term care is described in the MoH guidelines (Linee di indirizzo per l'assistenza alle persone in Stato Vegetativo e Stato di Minima Coscienza, http://www.salute.gov.it/imgs/C_17_pubblicazioni_1535_allegato.pdf, Accessed June 20, 2016) but in our sample, seemed to be done by few patients (only 20%). The fact that changes in diagnosis were mainly found during the time spent in rehabilitation units is in line with previous literature (Estraneo et al., 2014). This point can be explained with different points of view: in rehabilitation, the frequency of assessment is higher than in long‐term care units and so the probability to find an improvement is quite high also considering the earlier time from acute event. Another possible view is related to death rate: as we reported not all patients are still alive in the follow‐up time in our survey so we can collect data on the diagnostic changes in long‐term patients only in a smaller sample of patients respect to those analysed for rehabilitation. Moreover, considering patients’ death percentage, LOS in RH should be inserted in a general design of pathways of care. Data from Apulia region seem emblematic because a high death percentage value was found, although LOS in RH was quite high. An ad hoc analysis revealed that most of those patients died during the long‐term care phase and this could be related to the fact that in Apulia region there are no long‐term nursing homes dedicated to patients in VS and MCS as well as there are no tailored assistance protocols for patients at home (Linee di indirizzo per l'assistenza alle persone in Stato Vegetativo e Stato di Minima Coscienza, http://www.salute.gov.it/imgs/C_17_pubblicazioni_1535_allegato.pdf, Accessed June 20, 2016). This result entails a serious reflection on data interpretation: LOS in RH could be important and we can reflect on how many days could be spent for rehabilitation but if LOS in RH are not considered as one variable in a set of variables (Formisano et al., 2017) related to all phases of pathway of care, we cannot find an “equilibrium point” that really matches an appropriate pathway of cure and care for patients in VS and MCS. Another important point was also related to decision on discharge from rehabilitation units. In fact, according to national guidelines (Linee di indirizzo per l'assistenza alle persone in Stato Vegetativo e Stato di Minima Coscienza, http://www.salute.gov.it/imgs/C_17_pubblicazioni_1535_allegato.pdf, Accessed June 20, 2016), discharge is possible when the individualized rehabilitation program is completed (no variation in conscious state was shown, absence of severe respiratory failure, etc.). However, the application of clinical guidelines is very heterogeneous because they are subordinated to regional welfare policies that are very different across regions (e.g., some regions limited the hospitalization time in rehabilitation unit) and this could affect the LOS in RH value as well as the number of beds available in chronic facilities in each region.

Results on “changes in the diagnosis”, reported in Table 3, highlighted that a diagnosis of MCS or severe disability at rehabilitation discharge seemed to be important for avoiding patients worsening in long‐term care phase. Although this result could be influenced by the fact that worsening from VS was only death in our analysis, this result suggested paying attention to rehabilitation protocols demonstrating improvement in clinical status (diagnosis changed mainly during rehabilitation phase), in addition to time spent in a rehabilitation unit.

Finally, the number of caregivers/per patient had a significant role associated with prevention of worsening in clinical status and this is in line with previous literature in which the role of caregivers was important also to detect first signs of improvement in cognitive status of patients during clinical assessment. This could be associated with the fact that higher number of caregivers guarantee an accurate monitoring of patients around the 24 hr and caregivers are usually the first persons who note signs of recovery (Sattin et al., 2014). Moreover, these results should be interpreted considering that most of the patients were at home during their long‐term phase in our pilot study, and so the possibility for the main caregiver to have a support from other persons was fundamental both for physical health as well as for avoiding a too heavy emotional burden, a common risk for caregivers as reported in several articles (Corallo et al., 2015; Giovannetti, Leonardi, Pagani, Sattin, & Raggi, 2013; Leonardi, Giovannetti, Pagani, Raggi, & Sattin, 2012).

In the second area, one of the main problems reported by patients’ relatives was the need to have information on patients clinical status (Leonardi et al., 2012), especially in the early phases of the pathway of care. The difficulty to accept the situation for caregivers as well as the difficulty to define a clear prognosis could explain the negative (or the no evaluation) answer for this item of the questionnaire (DeVoe, Wallace, & Fryer, 2009; Dou, Gao, Lu, & Chang, 2014; Olding et al., 2016; Tsetsou, Oddo, & Rossetti, 2013). Regarding postacute and long‐term phases, the presence of nongovernment associations composed of relative of persons with DOC was required by several caregivers who often considered, as a weak point, NGOs absence. In Italy there were almost 40 associations for persons in VS and MCS at the moment, all working with professionals to develop good clinical practice guidelines and supporting caregivers of new persons in VS and MCS during the care process. The almost complete absence of psychologists in long‐term care phase was not evaluated as a strong point too. This result is very critical considering that previous studies highlighted high level of anxiety and depression in caregivers (Chiambretto & Vanoli, 2006; Pagani, Giovannetti, Covelli, Sattin, & Leonardi, 2014). The absence of services for emotional support could be critical both for those caregivers who were alone during their duties and for patients too. Finally, with regard to caregivers’ knowledge of the national and international legislation that applies to the care of persons with DOC, the relatively low levels of awareness of the UN Convention on the Rights of Persons with Disabilities (65.7% of respondents reported that they did not know it) and of NPCM‐DOC (only 25.7% of respondents were aware of the national agreement) highlighted by the INCARICO questionnaires is striking. Given the almost absolute lack of autonomy and complete dependence that characterize persons with DOC, it is vital that their caregivers are aware of the national and international Conventions and regulations, which identify the rights of people with severe disabilities and appropriate pathways of care, in order to promote equity in accessing healthcare and social services for persons with DOC.

Some limitations need to be taken into account. First, data on clinical status and severity were only indirectly considered in our study. Age, time from acute event, and LOS in first hospitalization in ICU units were used as covariates in our pilot analysis. However, association between information on care pathways and patients’ clinical outcomes data is complex, considering the huge number of variables that could influence the health care process. For example, etiology could be an important variable associated to high LOS values but we did not collect information on it in order to concentrate more on the care pathways reconstruction. In fact, we tried to increase caregivers’ compliance using a semistructured interview on this main issue, limiting the time dedicated to the interview. We know that the relationship between clinical status and LOS should be carefully analyzed in future research. Second, the last diagnosis collected for patients who were at home were those reported by multidisciplinary teams that evaluate each patient every year to confirm diagnosis providing public health assistance and devices. Unfortunately, we were not able to verify how each teams evaluated the patients and what kind of tools were used, although the multidisciplinary teams were composed of professionals from different medical area and a lot of patients reported that they require follow‐up medical visits periodically with expert professionals to monitor patients’ clinical status. Third, this study did not collect information on what kind of interventions caregivers required specifically (e.g., caregivers reported the lack of psychologists in chronic facilities but no data on what they requested were collected). Future studies are needed to analyze this issue. Moreover, in the present paper, we used a semistructured interview methods for population survey. As known, this approach implies that not all information can be checked and authors have to consider some answer received as true for definition. For example, the number of caregivers available to care patients were reported by main informal caregivers and we have no data to verify this information in our study. Finally, this study considers few data, from only 10 regions, in order to offer a new perspective and a starting point useful to all European countries to analyze the relationships between public health data, rehabilitation models, and patients’ outcomes. However, future studies are needed including a monitoring with standardized clinical scale scores and a larger sample than the one involved for this pilot research in order to compare different models.

## 5 CONCLUSIONS

The present pilot study highlights that there are differences in health care pathways models, despite the common national pathway of care for patients in VS and MCS. The role of days spent in rehabilitation units and the number of caregivers caring patients seems to be important variables for the relationship between health care pathways and clinical outcome although future public health considerations are needed. Moreover, caregivers reported needs to improve services supporting them during all the care process.

## DECLARATION OF INTEREST

The authors declare that they have no conflict of interest and the study gained the approval from the Fondazione IRCCS Istituto C. Besta Ethics committee and there are no constraints on publishing.

## AUTHOR CONTRIBUTIONS

*Study conception and design:* Sattin D., Morganti L., De Torres L., Leonardi M.; *Acquisition of data:* Sattin D., Dolce G., Arcuri F., Estraneo A., Cardinale V., Piperno R., Zavatta E., Formisano R., D'Ippolito M., Vassallo C., Dessi B., Lamberti G., Antoniono E., Lanzillotti C., Navarro J., Bramanti P., Corallo S., Zampolini M., Scarponi F., Avesani R., Salvi L., Ferro S., Mazza L., Martinuzzi A., Buffoni M., Leonardi M.; *Analysis and interpretation of data:* Sattin D., Morganti L., De Torres L., Leonardi M., Fogar P. Feller S., De Nigris F., Pessina A., Corsico P. *Drafting of manuscript:* Sattin D., Morganti L., De Torres L., Leonardi M.; *Critical revision:* Dolce G., Arcuri F., Estraneo A., Cardinale V., Piperno R., Zavatta E., Formisano R.,. D'Ippolito M., Vassallo C., Dessi B., Lamberti G., Antoniono E., Lanzillotti C., Navarro J., Bramanti P., Marino S., Zampolini M., Scarponi F., Avesani R., Salvi L., Ferro S., Mazza L., Martinuzzi A., Buffoni M., Leonardi M.; Fogar P. Feller S., De Nigris F., Pessina A., Corsico P.

## Supporting information


** **

** **
