# Supplementary figures and images for: Care pathways models and clinical outcomes in Disorders of consciousness
Source: Brain Behav. 2017 Jul 21;7(8):e00740. doi: 10.1002/brb3.740 (PMC5561306; doi:10.1002/brb3.740)

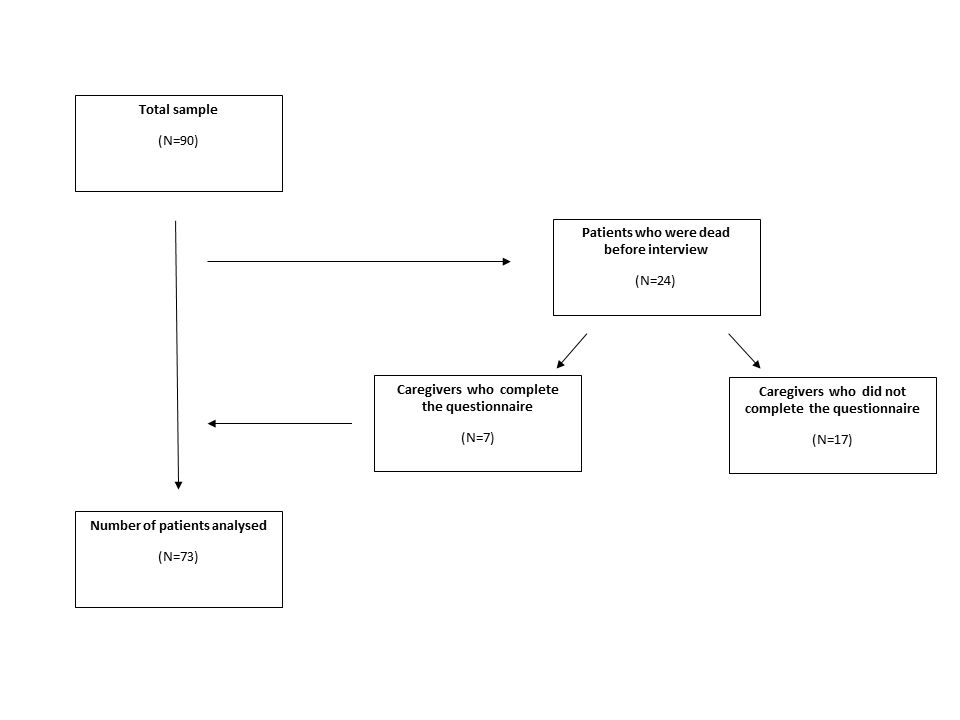

Supplement: Supplementary file 1 [file BRB3-7-e00740-s001.jpg]
