# Supplementary material for: Care pathways models and clinical outcomes in Disorders of consciousness
Source: Brain Behav. 2017 Jul 21;7(8):e00740. doi: 10.1002/brb3.740 (PMC5561306; doi:10.1002/brb3.740)
Supplement: Supplementary file 2 [file BRB3-7-e00740-s002.docx]

**Supplementary Materials**

**Description of the Italian health care models for persons with Disorders of consciousness.**

Italian model describes all the steps of health care process for patients with DOC. In details, after entering to an emergency department, patients with a severe brain injury (due to trauma, hypoxia, or cardiovascular aetiology or other) are admitted to Intensive care units or Neurosurgery units (if required) for few weeks. Then, the national model suggested a second phase in which patients who are clinically unstable, but do not require continue intensive care, could be admitted in tailored sub-acute unit, in order to start early rehabilitation treatments. After this step, patients should be hospitalized in neuro-rehabilitation units with professionals experienced in clinical management of severe brain injuries sequelae. In these units, if a patient quickly recovers cognitive and motor abilities, the model suggested that he/she has to follow a short-term intensive rehabilitation program (with length of hospital stay measured in weeks), otherwise a slow-to recover patient should receive long–term comprehensive rehabilitation (with a length of hospital stay measured in months). Finally, the Italian model indicates solutions for patients who remain in chronic stabilized unresponsive state, indicating tailored units in nursing homes and reporting example of “at home” projects for sustaining return to home.

**Description of Statistical analysis - Regression models**

Two multinomial multivariate logistic regression analysis were performed independently for acute/rehabilitation phase and long term phase following three steps (model A and B, model C and D respectively): in the first step one model including all variables was analysed and statistical significant of the model was tested. Then, if p-value of the model was significant, as second step, a backward stepwise methodology was performed to evidence variables that seemed statistically related to probability of selected outcome. A third step was performed only if no results emerged from the first two steps and a series of models were analysed including variables that showed a statistically significant p-value in univariate analysis and then testing their relationship with others. Independent variables were divided in variables related to acute/rehabilitation phase and variables of long-term care phase in relation to the general care pathway phase (e.g. total length of hospital stay or adherence with regional care pathway were variables related to the beginning phase of care, when a patient often moved from one hospital unit to another one, and not to the long term care phase when patients are at home or admitted in a nursing home with a definition of “patients with a chronic condition”). Two variables, time from acute event and age, were included in regression analysis, in order to verify relationships with outcomes.

Dependent variable: clinical improvement plus stable situation for acute/rehabilitation phases whereas clinical worsening for long term care phase.

**Table 1 SM – Univariate binary Logistic regression analysis: relationships between improving in clinical status and variables related to acute and post acute phases.**

|  |  |  |  | **INDIPENDENT VARIABLES** | | | | | |  |
| --- | --- | --- | --- | --- | --- | --- | --- | --- | --- | --- |
|  |  |  |  | **Time from acute event** | **Age** | **LOS - ICU** | **LOS – SA+RH** | **APC** | **TP** |  |
| **UNIVARIATE ANALYSIS** |  |  |  |  |  |  |  |  |  |  |
| **Y=Improving+clinically stable** |  | B(SE) |  | .026(.021) | -007(0.12) | -.001(.006) | **.005(.001)** | -.859(.829) | -.048(.118) |  |
|  |  | OR  (95% CI) |  | 1.026  (.986-1.068) | .993  (.970-1.018) | .999  (.987-1.012) | **1.005**  **(1.002-1.008)** | .424  (.083-2.149) | .953  (.757-1.200) |  |
|  |  | Sig. |  | .212 | .595 | .904 | **.001**** | .300 | .681 |  |

**Table 2 SM - Univariate binary Logistic regression analysis: relationship between worsening in clinical status and variables related to long term care phase.**

|  |  |  |  | **INDIPENDENT VARIABLES** | | | | |  |
| --- | --- | --- | --- | --- | --- | --- | --- | --- | --- |
|  |  |  |  | **Diagnosis** | **nCG** | **Time from acute event to admission in nursing home/home** | **Transfer from nursing home/home to ICU** | **Last admission** |  |
|  |  |  |  | Other  diagnosis |  |  |  | At home |  |
| **UNIVARIATE ANALYSIS** |  |  |  |  |  |  |  |  |  |
| Y=worsening |  | B(SE) |  | -.680  (.490) | -1.493  (.348) | .006  (.046) | .441  (.554) | 1.600  (.539) |  |
|  |  | OR  (95% CI) |  | .507  (.194-1.325) | .225  (.114-.444) | 1.006  (.919-1.100) | 1.554  (.525-4.603) | 4.995  (1.724-14.242) |  |
|  |  | Sig. |  | .166 | .000*** | .902 | .426 | .003** |  |

**The INCARICO Questionnaire.**

Questionnaire was composed by three sections.

The first section collected information related to caregiver, such as sociodemographic data, hours dedicated to care, number of persons available for caring, and their knowledge on laws regulating care pathways for patients with DOC;

In the second part, items focused on patients and caregiver was asked to describe the entire pathway of care done by the patient they care for: identification of each clinical unit that hospitalized the patient until phone interview, length of hospital stay (LOS) data and information on diagnosis at admission and discharge of each unit were collected.

In the last part of the questionnaire, researchers asked the caregiver to evaluate services and degree of technical information received by clinicians as well as professionals communication modalities in acute, post-acute and long term care phases using close-ended interview questions. Moreover, the questionnaire collected information on involvement of caregiver during care process, opinions on quality and quantity of services the patients received and waiting time for admission in rehabilitation or long term care units. Each item of the questionnaire was chosen in according to NGOs collaborating to INCARICO and based on results found in different previous Italian studies. This part of the questionnaire is presented below.

**Questionnaire - Caregivers opinions on care process of persons with Disorder of consciousness.**

Considering the whole patients care process, please indicate the weak (if any) and the strong (positive) aspects found in every stage of the pathway of care (acute, rehabilitation, long-term care). Starting from the acute phase, which were the principal positive aspects? And which were the most critical ones that obstructed the care pathway?

(*Note for the interviewer: Initially the interviewer should let the caregiver talk spontaneously and try to identify the corresponding item from the table below and/or add the new themes reported by caregiver. Then the interviewer should verify that opinion on every items was asked to the caregiver, otherwise the missing ones should be read. Please remember that the principal goal is to get the overall impression about a certain phase. Therefore, if a patient was admitted to more than one centers during the same stage, the interviewer must consider only the overall impression about that specific phase of the care process and not about each single center.*)

| **ACUTE PHASE** | **Strong point / stage’s added value** | **Weak point / aspect to improve** | **Not evaluated** |
| --- | --- | --- | --- |
| Healthcare professionals’ communication modalities and information completeness (information about health condition and evolution) | ⃝ | ⃝ | ⃝ |
| Quality and quantity of the received healthcare services *(specify any perceived differences between quantity and quality)* | ⃝ | ⃝ | ⃝ |
| Possibility to decide together with healthcare professionals on the center for the next phase of care | ⃝ | ⃝ | ⃝ |
| Waiting time for admission in the center of the next phase of care | ⃝ | ⃝ | ⃝ |
| - | ⃝ | ⃝ | ⃝ |
| - | ⃝ | ⃝ | ⃝ |
| - | ⃝ | ⃝ | ⃝ |
| - | ⃝ | ⃝ | ⃝ |

| **POST-ACUTE PHASE (REHABILITATION)** | **Strong point / stage’s added value** | **Critical point / aspect to improve** | **Not evaluated** | **N/A** |
| --- | --- | --- | --- | --- |
| Center’s reception modalities (services charter, brochures, meetings) at admission or later | ⃝ | ⃝ | ⃝ | ⃝ |
| Healthcare professionals’ communication modalities and completeness of information (information about health condition and evolution) | ⃝ | ⃝ | ⃝ | ⃝ |
| Visiting policies (i.e. out-of-schedule visits, extended visiting hours, etc.) | ⃝ | ⃝ | ⃝ | ⃝ |
| Families Support Service (Psychologist) | ⃝ | ⃝ | ⃝ | ⃝ |
| Families Support Service (Social Worker) | ⃝ | ⃝ | ⃝ | ⃝ |
| Families Support Service (Voluntary associations) | ⃝ | ⃝ | ⃝ | ⃝ |
| Setting quality and comfort (i.e. chances to go outdoor, common areas to socialize) | ⃝ | ⃝ | ⃝ | ⃝ |
| Possibility to decide together with healthcare professionals on the center for the next phase of care | ⃝ | ⃝ | ⃝ | ⃝ |
| Waiting time for admission in the center of the next phase of care | ⃝ | ⃝ | ⃝ | ⃝ |
| Rehabilitation service’s quality in terms of rehabilitation project’s multidisciplinarity and healthcare professionals’ training (presence of highly qualified staff) | ⃝ | ⃝ | ⃝ | ⃝ |
| Rehabilitation service’s quantity (mean time of physiotherapy, nurse care, medical specialization, cognitive therapy– please specify for each service whereas possible) | ⃝ | ⃝ | ⃝ | ⃝ |
| - | ⃝ | ⃝ | ⃝ | ⃝ |
| - | ⃝ | ⃝ | ⃝ | ⃝ |
| - | ⃝ | ⃝ | ⃝ | ⃝ |
| - | ⃝ | ⃝ | ⃝ | ⃝ |

| **LONG-TERM CARE PHASE** | **Strong point / stage’s added value** | **Critical point / aspect to improve** | **Not evaluated** | **N/A** |
| --- | --- | --- | --- | --- |
| Center’s reception modalities (services charter, brochures, meetings) at admission or later | ⃝ | ⃝ | ⃝ | ⃝ |
| Healthcare professionals’ communication modalities and completeness of information (information about health condition and evolution) | ⃝ | ⃝ | ⃝ | ⃝ |
| Visiting policies (i.e. out-of-schedule visits etc.) | ⃝ | ⃝ | ⃝ | ⃝ |
| Families Support Service (Psychologist) | ⃝ | ⃝ | ⃝ | ⃝ |
| Families Support Service (Social Worker) | ⃝ | ⃝ | ⃝ | ⃝ |
| Families Support Service (Voluntary associations) | ⃝ | ⃝ | ⃝ | ⃝ |
| Setting quality and comfort (i.e. chances to go outdoor, common areas to socialize) | ⃝ | ⃝ | ⃝ | ⃝ |
| Assistance in case of urgency/emergency | ⃝ | ⃝ | ⃝ | ⃝ |
| No bed’s loss in case of readmission or recovery in rehabilitation centers for patient in long-term care | ⃝ | ⃝ | ⃝ | ⃝ |
| Possible readmission in rehabilitation centers due to medical issues or the need to redefinition of the rehabilitation plan (i.e. botulin toxin, intrathecal baclofen pump implantation or modulation, neurophysiological examination, etc.) | ⃝ | ⃝ | ⃝ | ⃝ |
| Rehabilitation service’s quality in terms of rehabilitation project’s multidisciplinarity and healthcare professionals’ training (presence of highly qualified staff) | ⃝ | ⃝ | ⃝ | ⃝ |
| Rehabilitation service’s quantity (mean time of physiotherapy, nurse care, medical specialization, cognitive therapy– please specify for each service whereas possible) | ⃝ | ⃝ | ⃝ | ⃝ |
| - | ⃝ | ⃝ | ⃝ | ⃝ |
| - | ⃝ | ⃝ | ⃝ | ⃝ |
| - | ⃝ | ⃝ | ⃝ | ⃝ |
| - | ⃝ | ⃝ | ⃝ | ⃝ |
